# Daily gender expression is associated with psychological adjustment for some people, but mainly men

**DOI:** 10.1038/s41598-021-88279-4

**Published:** 2021-04-27

**Authors:** Adriene M. Beltz, Amy M. Loviska, Alexander Weigard

**Affiliations:** Department of Psychology, University of Michigan, 2227 East Hall, 530 Church Street, Ann Arbor, MI 48109 USA

**Keywords:** Human behaviour, Preclinical research

## Abstract

To what extent does gender expression vary day-to-day? Are daily changes related to psychological adjustment in the same way for all individuals? A person-specific approach was used to answer these questions in a 75-day intensive longitudinal study. Fifty-seven cisgender adults (27 women) provided over 4000 reports of daily masculinity and femininity and of three indices of internalizing problems. Results revealed: (a) substantial daily fluctuations in gender expression, especially in women; (b) sample-level links between daily increases in femininity or reductions in masculinity and heightened anxiety, depression, and self-reproach for men, but no apparent links for women; and (c) person-specific links between gender expression and psychological adjustment, such that some women reported internalizing problems with reduced masculinity (average male pattern) and some men reported problems with heightened masculinity (opposite the average male pattern). Findings highlight how intensive longitudinal research can illuminate the uniqueness of gender-related daily experiences, and their implications for the wellbeing of *individuals*.

## Introduction

Questions about the nature of masculinity and femininity have long been asked in scientific investigation and public conversation. Today, it is clear that gender expression is a continuum. This is reflected in basic and applied research^1–4^ as well as in cultural norms (e.g., increased availability of gender-inclusive restrooms and acknowledgment of gender-inclusive titles on official forms). Much less clear, however, is the extent to which gender expression fluctuates along this continuum—as life roles change, with daily experiences, and during momentary interactions. The aim of this study was to examine day-to-day fluctuations in gender expression, and their daily associations with psychological adjustment, using 75-day intensive longitudinal data and person-oriented analytic approaches.

### Gender expression

Gender expression is the way in which an individual enacts their thoughts and beliefs about their gender, or their gender self-concepts, such as in the femininity and masculinity of their appearance and behavior. It is a combination of an individual’s own view of their gender identity and their knowledge of, and prescription to, sociocultural gender norms, expectations, and attitudes. Moreover, gender is multidimensional, and thus, expressed masculine and feminine self-concepts are also related to sexual development, gender stereotypes, personality qualities, roles, orientations, ideologies, compatibility, and pressure, among other gendered psychological phenomena^5–11^. Thus, masculinity and femininity are biopsychosocial constructs that reflect an individual’s sense of themselves as (none or some degree of) male and/or female that unfolds in an environment that (to some degree) distinguishes girls and women from boys and men^8,11–13^.

Gender is often assumed to be stable; masculinity and femininity are even considered to be *traits* by some scholars^6,9,11,14^. There is, however, evidence of change. Gender self-concepts form in early childhood^15^, undergo questioning and shifts in adolescence^7,9,16^, and are marked by increases in expressed femininity for both men and women in late adulthood^8,17,18^. Gender expression also changes during rapid life transitions. For instance, femininity increases for both mothers and fathers with the birth of a first child^19^, and women who have had hysterectomies and mastectomies report decreases in femininity^20^.

Recently, there have been calls for increased investigation into the fluidity of gender, including short-term changes in expressed masculinity and femininity^21–23^. For example, children’s gender typicality fluctuates during free play, as they engage with different toys, activities, and peers^24^. In experimental settings, adolescent males and females report greater femininity when playing a game with a female partner, while young women report greater femininity when playing the same game with a male partner^25,26^. These findings generalize to the “real-world”: Young men report feeling more feminine when interacting with female peers, and both young men and women report feeling more masculine when interacting with male peers^27^. Although research is limited, there is clearly evidence for both long-term and context-dependent fluctuations in gender expressions.

### Gender expression and psychological adjustment

Generally, individuals who feel typical for their sex (i.e., females with high femininity and males with high masculinity) tend to report better psychological well-being. Across childhood, adolescence, and young adulthood, individuals who feel less typical for their sex or less content with their gender report low self-worth as well as high depression and negative affect^7,24,28–32^. Underlying mechanisms have not been comprehensively studied, but it appears that part of the relation between gender non-conformity and depression may be explained by bullying, and that part of the relation between gender typicality and self-worth may be explained by communality, or feelings and displays of social closeness^30,31^. Thus, feelings of typicality seem to be important for psychological well-being.

There is, however, a paucity of information concerning links between short-term fluctuations in gender expression and psychological adjustment, or the degree to which individuals experience internalizing (e.g., anxiety and depression) and externalizing (e.g., conduct disorder and substance use) problems. If gender expression changes over time, does psychological adjustment change, too? One study provides early evidence: Girls who were more flexible in their gender expression when playing with other girls and when playing with boys had generally higher positive emotion and fewer externalizing problems than those who were less so^24^. This insight was afforded by unique time series data, consisting of 64–212 coded observations per child, and suggests that investigations of gender-related fluctuations and moment-to-moment links with psychological adjustment require extensive within-person assessments.

### Person-specificity of gender expression

Within-person, intensive longitudinal data (in which many assessments are collected from the same individuals over time) are also well-suited to address another significant limitation of past work: the assumption that gender expression has similar psychological correlates for all women and all men. Although heterogeneity, or meaningful between-person variability, in gender-related phenomena is acknowledged in theory^6,8^, homogeneity or between-person similarity is typically assumed in statistical analyses and inferences in gender research (similar to research on other phenomena^33^). Indeed, the missed opportunity to study the person-specific nature of gender was recognized over three decades ago: “The fundamental logical error that seems to have been made is to assume that an aggregation of statistical facts distinguishing between two groups of individuals, in this instance men and women, can automatically be combined to arrive at portraits of the typical member of each group…Most men and women exhibit a fair number of gender-congruent characteristics, but the particular assortment varies widely from one man or woman to the next” [9, p. 77]. Unfortunately, little empirical progress in individualizing models of gender—let alone its links with psychological adjustment—has been made since this astute observation, potentially due to challenges in acquiring and analyzing the intensive longitudinal data necessary for personalization.

### Current study

The aim of this study is to answer three questions: (1) To what extent does gender expression vary on a day-to-day basis? (2) Are daily fluctuations in expression associated with daily fluctuations in psychological adjustment, particularly internalizing problems? and (3) Do the answers to these questions differ from person-to-person; in other words, does daily gender expression fluctuate for some people more than others in ways differentially associated with internalizing problems? This was accomplished utilizing data from a 75-day intensive longitudinal study of self-identified men and women. We hypothesized that gender expression would fluctuate across days, and that daily expression would be linked to daily adjustment, such that increases in sex-congruent expressions (e.g., masculinity for men and femininity for women) would be associated with reductions in psychological adjustment problems. Finally, we expected person-specificity in links between gender expression and psychological adjustment, or that the sample-level expression-adjustment pattern would not apply to every participant.

## Methods

Data are from a larger intensive longitudinal study on sex hormones and behavior. Subsets of data on cognition^34^, personality^35^, and affect^36^ have been previously reported. Original data on daily gender expression as well as anxiety, depression, and self-reproach are included here. The University of Michigan Institutional Review Board—Health Sciences and Behavioral Sciences approved this research, which was conducted according to its guidelines.

### Participants

Participants were 57 adults, including 30 self-identified men and 27 self-identified women with a regular, natural menstrual cycle; none were using psychotropic or sex hormone-containing medications. Participants were between 18 and 38 years old (*M* = 21.91; *SD* = 3.93) and were White/Caucasian (58%), Asian (33%), Black/African American (7%), or multiple races (2%) and predominately non-Hispanic (95%). Men and women did not differ in age, *t*(55) = − 0.46, *p* = 0.651, race, *χ*^2^(3) = 4.28, *p* = 0.233, or ethnicity, *χ*^2^(1) = 0.913, *p* = 0.339. Daily response rates for the measures reported were between 81 and 100% (*M* = 94%; *SD* = 4.29).

Participants represent a subset of the 235 participants recruited for an intensive longitudinal study via university-sponsored subject pools, email blast lists, and databases as well as via flyers placed in the surrounding community. Most were not included in this study because they either were using exogeneous hormones (*n* = 86 female oral contraceptive users would reduce power to detect effects in men) or completed less than 80% of the daily assessments for the measures reported here (*n* = 92); this cutoff is based on measure validation in this sample and missing data thresholds in psychological time series^34,37^.

### Procedures

Following a laboratory session in which informed consent was obtained and baseline testing was done, participants completed 20-min daily assessments of emotion, behavior, personality, and cognition every evening for 75 days. The assessments were administered through Qualtrics (with a unique link emailed each night at 5:00 p.m.) and could be completed on any Internet-capable device after 8:00 p.m. or before bedtime; links expired at 12:00 p.m. the next day. Participants received up to $215 for their efforts: course credit or $15 for the laboratory session and $200 for the 75 daily assessments, which was tiered based on the number of completed assessments. Specifically, participants received $1 for each completed assessment. If they completed at least 80% of the assessments, then their compensation increased to $2 per assessment, and if they completed at least 90%, then they received a $50 bonus. After 30 days, participants whose completion rates fell below 50% were withdrawn from the study.

### Measures

In each of the 75 daily assessments, participants completed valid and reliable measures of gender expression and internalizing problems. The measures were modified so that participants responded about their thoughts, feelings, and behaviors with respect to the past 24 h (instead of with respect to themselves in general).

**Daily gender expression** was assessed with the Sex Role Identity Scale^10^, which is a six-item self-report measure of self-perceived masculine and feminine expressed self-concepts. Participants were asked how masculine and then how feminine they looked, acted, and felt in general during the past day; they responded on a 5-point Likert scale (1 = “Not at All” to 5 = “Extremely”). Importantly, “masculine” and feminine” were not defined for participants based on sociocultural gender norms, nor were traits stereotypically associated with masculinity and femininity listed for participant endorsement. Instead, participants were simply asked, for example, “*In general, how masculine do you think you were today*?” and they responded based on what “masculine” means to them.

After reverse coding the three feminine items, all items were averaged to create a bipolar, unidimensional gender expression score ranging from 1 (maximum self-perceived femininity) to 3 (equal masculinity and femininity) to 5 (maximum self-perceived masculinity). This unidimensional scoring approach is consistent with current continuum-based theories of gender identity^2,4^, empirical research on gender expression^1,3^, and statistical evidence that masculine and feminine self-concepts are highly inversely related, with stronger links to psychological adjustment when combined than when separated into two factors^7,9^. Several classic theories of gender self-concept, however, purport that masculinity and femininity are separate dimensions, consistent with sociocultural norms for the appearance and behavior of men and women^6,14^. Although these theories generally concern gendered personality characteristics, and thus, are only partially related to gender expression, separate three-item masculine and feminine subscales were also created (using averages) and used in parallel analyses reported in Supplementary Information.

**Daily anxiety, depression, and self-reproach** were assessed with subcomponents from the NEO Five Factor Inventory, which is a 60-item self-report measure^38,39^. Participants were asked to indicate their agreement with statements on a 5-point Likert scale (1 = “Strongly Disagree” to 5 = “Strongly Agree”). Specifically, anxiety, depression, and self-reproach were rationally-derived from the 12-item Neuroticism subscale; they were created with the a priori goal of distinguishing among finer grained facets of the construct, especially anxiety and depression^39^. The rational subscales were found to be reliable (with Cronbach’s alphas ranging from 0.70 to 0.78^39^), valid (e.g., with the anxiety subscale correlating highly with an established anxiety measure but only moderately with an established depression measure^39^), and meaningful adjustment indices in psychological studies^36,40^.

### Analysis plan

Three sets of analyses were conducted; Type I error was set at 0.05 for all analyses. First, daily gender expression fluctuations were estimated for each person by calculating the intraindividual standard deviations (iSDs) of their feminine-to-masculine continuum scores across the 75 days. This is a composite that reflects an individual’s variability in expression with respect to their own average. Standard deviations at or close to zero reflect little variability across days, and standard deviations greater than zero reflect person-specific variability in gender expression. Thus, one-sample *t*-tests were used to test whether iSDs for the full sample significantly differed from zero. Analyses were also conducted separately for and comparing men (coded 1) and women (coded 0). Within-person variation in gender expression was contextualized by comparing it to between-person variation in expression. Specifically, the mean feminine-to-masculine iSD across participants was divided by the sample standard deviation of mean feminine-to-masculine expression across days (i.e., *M*_*iSD*_/*SD*_*M*_). Finally, analyses were repeated using masculinity and femininity as separate indices to ensure the feminine-to-masculine continuum of gender expression did not obscure potential categorical effects.

Second, links between daily gender expression and psychological adjustment were estimated for the full sample, while accounting for repeated assessments within participants, using multilevel models; individual differences linked to gender were also examined. In three models (each with a different outcome: anxiety, depression, and self-reproach), gender was a time-invariant predictor, and day (centered at 0), gender expression (i.e., grand mean centered feminine-to-masculine continuum), and the interaction between gender expression and gender were time-varying predictors. A random intercept and random slopes for day and gender expression were estimated with unstructured error covariance using restricted maximum likelihood (REML). Significant effects for gender expression or its interactions with gender indicate that daily expression is associated with daily psychological adjustment in different ways for men and women. Interactions were probed with simple main effects analyses (i.e., running separate models for men and women). Multilevel models were also repeated using masculinity and femininity as predictors in separate models to reveal potential categorical effects.

Third, the person-specificity of daily links between gender expression and psychological adjustment were examined using within-person residualized correlations. Leveraging the intensive longitudinal design, this determines the extent to which findings from the multilevel models, which include within- and between-person effects, generalize to individuals^41^. For each person, pairwise correlations of daily gender expression and each internalizing outcome were estimated. Because daily data are not independent, first order temporal dependencies were removed from each variable prior to the correlations^42^. For each participant, each variable was regressed on its own scores from the previous day, and residuals were used in correlations. These correlations reveal the daily synchrony between gender expression and psychological adjustment for an individual. Participants were then classified according to whether their correlation *magnitudes* were meaningful (i.e., greater than a small effect size of *r* = 0.10^43^), and whether the correlation *directions* matched expectations that sex-congruent gender expressions were associated with reductions in internalizing problems (i.e., inverse feminine-to-masculine relations for men, but positive feminine-to-masculine relations for women).

## Results

After reporting gender differences in all study variables (averaged across 75 days), analyses addressing study aims are presented. Specifically, daily fluctuations in gender expression, sample-level links between daily expression and daily psychological adjustment (i.e., anxiety, depression, and self-reproach), and person-specific links between daily expression and adjustment are reported in sequence. Results for the feminine-to-masculine continuum are reported here, and results for the separate masculine and feminine dimensions, which provided similar conclusions, are in the Supplementary Information.

### Gender differences

**Figure 1:**
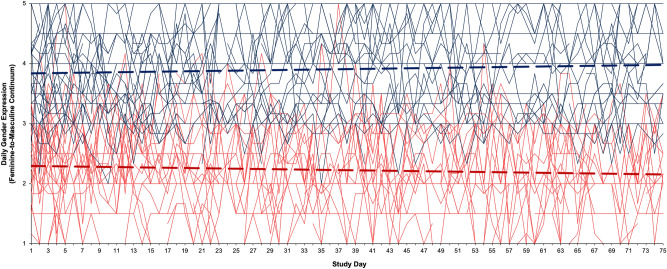
Line graph of daily gender expression (feminine-to-masculine continuum) for individual men (thin blue lines) and women (thin red lines) across the 75 days of the intensive longitudinal study. Thick dashed lines show trends averaging across participants of the same self-reported gender.

Mean gender expression in the full sample was at the midpoint of the measure (*M* = 3.10, *SD* = 1.07), and the expected gender difference in the mean daily feminine-to-masculine continuum was found, *t*(55) = − 9.80, *p* < 0.001, *d* = 2.63, with men (*M* = 3.91, *SD* = 0.69) having scores in the masculine range (> 3) and women (*M* = 2.21, *SD* = 0.60) having scores in the feminine range (< 3). This difference can be seen in Fig. 1; the dashed trend lines show that the average gender expression scores for men (in blue) are consistently higher than the reports for women (in red) across the 75 days of the study.

There were no gender differences in mean internalizing problems across the 75 days of the study. Men and women were similar in anxiety, *t*(55) = − 0.33, *p* = 0.741, *d* = 0.09, depression, *t*(55) = − 0.67, *p* = 0.509, *d* = 0.18, and self-reproach, *t*(55) = − 0.53, *p* = 0.598, *d* = 0.15. Sample averages for anxiety (*M* = 2.62, *SD* = 0.68), depression (*M* = 2.46, *SD* = 0.66), and self-reproach (*M* = 2.06, *SD* = 0.67) were slightly below the scale midpoints of 3.

### Daily fluctuations in gender expression

There was substantial fluctuation in daily feminine-to-masculine expression, according to a one-sample *t*-test of iSDs, *t*(56) = 11.55, *p* < 0.001, *d* = 1.55, indicating that the sample average iSD of 0.31 (*SD* = 0.20) was greater than 0. Moreover, within-person variation in gender expression is nearly a third as large as the variation between people (i.e., standard deviation of feminine-to-masculine expressions across the sample): *M*_*iSD*_/*SD*_*M*_ = 0.31/1.07 = 0.29. Across individuals, gender expression iSDs ranged from 0 to 0.83, with 93% of the sample reporting fluctuation, but 4 individuals (1 woman and 3 men) reporting absolute stability; these individuals were excluded from the subsequent person-specific analyses (see “Person-specific links between daily gender expression and psychological adjustment” below). This is seen in the individual plots in Fig. 1; each thin line represents the reports for one individual, with the vast majority increasing or decreasing from day-to-day. Interestingly, however, the daily variation predominately occurs within gender-typical ranges, that is, most fluctuation for men occurs between values of 3 and 5, and most fluctuation for women occurs between values of 1 and 3.

When considered separately, both men, *t*(29) = 7.63, *p* < 0.001, *d* = 1.39, and women, *t*(26) = 9.21, *p* < 0.001, *d* = 1.76, had significant fluctuation in daily gender expression. When comparing the genders, however, women (*M* = 0.37, *SD* = 0.21) reported significantly greater fluctuations than men (*M* = 0.25, *SD* = 0.18), *t*(55) = 2.20, *p* = 0.032, *d* = 0.61. Consistent with this, within-person variation in gender expression was a third as large as the between-person variation among men (0.25/0.69 = 0.36), but nearly two-thirds as large as the between-person variation among women (0.37/0.60 = 0.62). This is also seen in the individual plots in Fig. 1: Daily reports for women are more often in the male-typical range (i.e., red lines above values of 3) than daily reports for men are in the female-typical range (i.e., blue lines below values of 3).

Results from analyses considering masculinity and femininity as separate dimensions are provided in the Supplementary Information. They are largely consistent with patterns seen in the feminine-to-masculine continuum. Daily fluctuations in both dimensions were significantly greater than 0 for the full sample as well as for men and women. Women had greater fluctuations than men in both femininity and masculinity, although the latter was not statistically significant (despite a small-to-moderate effect size, *d* = 0.33).

### Sample-level links between daily gender expression and psychological adjustment

Multilevel models predicting daily internalizing (i.e., anxiety, depression, and self-reproach) from assessment day, daily gender expression (feminine-to-masculine continuum), gender, and the interaction of gender expression and gender revealed significant and consistent fixed effects, as seen in Table 1. For all outcomes, the interaction between gender and gender expression was significant, indicating that the link between expression and psychological adjustment differed for men and women. In follow-up analyses probing the interaction in separate models for men and women, significant inverse associations between the feminine-to-masculine continuum and internalizing outcomes were found for men, but no significant associations existed for women. Thus, anxiety, depression, and self-reproach increased with more feminine/less masculine scores in men. Results from multilevel models considering masculinity and femininity as separate dimensions in Supplementary Table 1 and Table 2, respectively, are consistent with this pattern of results, such that increased anxiety, depression, and self-reproach are associated with both increased femininity and decreased masculinity in men, but that relations are not present for women.

**Table 1:** Multilevel model results for the sample-level link between daily gender expression (feminine-to-masculine continuum) and three indices of psychological adjustment by gender.

Outcome	Parameter or fit index	Unstandardized estimate (SE) or fit statistic		
		Full sample	Men	Women
Anxiety	**Fixed effects**			
	Intercept	2.68 (0.13)***	2.92 (0.12)***	2.67 (0.15)***
	Day	− 0.0006 (0.001)	− 0.0005 (0.001)	− 0.0007 (0.002)
	**Expression**	0.08 (0.06)	− 0.33 (0.07)***	0.07 (0.06)
	Gender	0.24 (0.18)	–	–
	Gender*Expression	− 0.41 (0.09)***	–	–
	**Random effects**			
	Intercept	0.42 (0.09)***	0.34 (0.11)**	0.55 (0.18)**
	Day	0.00005 (0.00001)***	0.00004 (0.00001)**	0.00006 (0.00002)**
	**Expression**	0.05 (0.02)**	0.07 (0.04)	0.05 (0.02)*
	**Model fit**			
	AIC	7884.91	3967.76	3918.96
Depression	**Fixed effects**			
	Intercept	2.47 (0.12)***	2.78 (0.10)***	2.45 (0.13)***
	Day	− 0.0006 (0.0007)	− 0.0004 (0.001)	− 0.0009 (0.0009)
	**Expression**	0.04 (0.06)	− 0.29 (0.08)**	0.02 (0.06)
	Gender	0.31 (0.16)	–	–
	Gender*Expression	− 0.34 (0.09)**	–	–
	**Random effects**			
	Intercept	0.31 (0.07)***	0.25 (0.08)**	.40 (.14)**
	Day	0.00002 (0.00001)**	0.00003 (0.00001)**	0.00001 (0.00001)
	**Expression**	0.07 (0.02)**	0.11 (0.05)*	0.04 (0.03)
	**Model fit**			
	AIC	7674.79	3928.26	3754.72
Self-reproach	**Fixed effects**			
	Intercept	1.94 (0.12)***	2.31 (0.13)***	1.93 (0.12)***
	Day	0.002 (0.001)	.0005 (.001)	0.003 (0.001)*
	**Expression**	0.03 (0.06)	− 0.27 (0.09)**	0.03 (0.05)
	Gender	0.39 (0.16)*	–	–
	Gender*Expression	− 0.31 (0.09)**	–	–
	**Random effects**			
	Intercept	0.32 (0.07)***	0.39 (0.12)**	0.31 (0.10)**
	Day	0.00004 (0.00001)***	0.00005 (0.00002)**	0.00003 (0.00001)**
	**Expression**	0.05 (0.02)*	0.15 (0.07)*	0.02 (0.02)
	**Model fit**			
	AIC	6542.00	3631.53	2910.71

Significant effects concerning gender expression (i.e., feminine-to-masculine continuum) are bold, and significant interactions in the full sample were probed with separate follow-up analyses in men and women. *p < 0.05; **p < 0.01; ***p < 0.001*.*

For many outcomes in the full sample and separate models for men and women, there were also significant random effects for the intercept, day, and gender expression. These indicate the presence of significant individual differences in baseline levels of anxiety, depression, and self-reproach and how they change over study days with respect to gender. There were similar patterns for random effects in the feminine-to-masculine continuum-based analyses of gender expression and in analyses considering masculinity and femininity as separate dimensions.

### Person-specific links between daily gender expression and psychological adjustment

Person-specific associations of feminine-to-masculine gender expression with anxiety, depression, and self-reproach (after variables were residualized for first order temporal dependencies) quantified individual differences in the daily synchrony between gender expression and internalizing problems. Table 2 shows descriptive statistics and correlations separately for men and women.

**Table 2:** Descriptive statistics for person-specific correlations between daily gender expression (feminine-to-masculine continuum) and three indices of psychological adjustment by gender.

Correlation	Men (*n* = 26)					Women (*n* = 26)				
	Range	*M*	*SD*	*r* > + 0.10	*r* < − 0.10^a^	Range	*M*	*SD*	*r* > + 0.10^a^	*r* < − 0.10
Expression and Anxiety	− 0.51, 0.19	− 0.12	0.18	15%	46%	− 0.18, 0.36	0.04	0.16	35%	27%
Expression and Depression	− 0.50, 0.19	− 0.10	0.19	19%	42%	− 0.32, 0.52	0.02	0.20	27%	27%
Expression and Self-reproach	− 0.50, 0.16	− 0.14	0.17	8%	54%	− 0.54, 0.36	− 0.02	0.18	23%	31%

Data from 4 men and 1 woman are missing due to 0 (or near 0) variations in daily gender expression. Data in the *r* > + 0.10 and *r* < − 0.10 columns reflect the percentages of individuals of a given gender who had correlations between gender expression and psychological adjustment that exceeded thresholds for small effects (Cohen, 1988).

^a^Indicates the hypothesized direction of correlation (i.e., sex-incongruent daily expression associated with problems).

For anxiety, correlations ranged from − 0.51 to 0.36 (*M* = − 0.04, *SD* = 0.19), with 62% meeting the threshold for a meaningful effect (i.e., percentages of participants with |*r*|> 0.10 averaged across men and women). There was an average effect of − 0.12 across men that was below the negative threshold for 46% of men (and as strong as − 0.51 in one individual) but was above the positive threshold for another subset (15%). The average effect was approximately null in women (0.04), with 35% reporting that feminine-to-masculine increases co-occurred with anxiety increases, but nearly as many (27%) reporting co-occurring anxiety decreases.

Similar results were found for person-specific gender expression correlations with depression and self-reproach. For depression, full sample correlations ranged from − 0.50 to 0.52 (*M* = − 0.04, *SD* = 0.20), with 58% meeting the threshold for a meaningful effect. In men, there was again a small average inverse effect that typified 42% of individuals, but in women, the average effect was basically null with 27% showing a positive relation between feminine-to-masculine expression and another 27% showing an inverse relation. For self-reproach, full sample correlations ranged from − 0.54 to 0.38 (*M* = − 0.08, *SD* = 0.19), with 58% having a meaningful effect. In men, the small average inverse effect typified a majority (54%) of individuals, and the average null effect in women was likely due to 23% showing a positive relation between feminine-to-masculine expression and another 31% showing an inverse relation.

**Figure 2:**
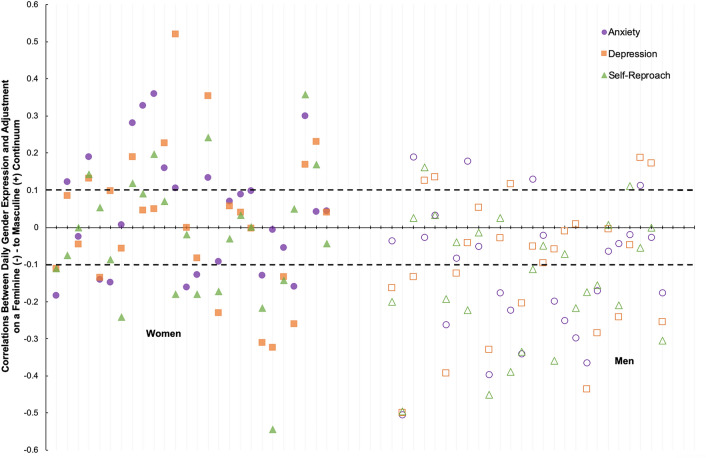
Scatterplot of person-specific correlations (residuals from first order autocorrelations) between daily gender expression (feminine-to-masculine continuum) and three indices of daily psychological adjustment: anxiety (purple circles), depression (orange squares), and self-reproach (green triangles). The y-axis depicts the strength of the correlations, and the x-axis depicts individual participants, with women (solid points) on the left and men (open points) on the right. Thick dashed lines at *r* = 0.10 and *r* = −0.10 demarcate a small effect size (Cohen, 1988), with correlations above and below the lines, respectively, reflecting meaningful individual-level correlations.

Person-specific residualized correlations between gender expression and psychological adjustment are visualized in Fig. 2. Each point shows a correlation for a different outcome for a unique individual (ticks on the x-axis), with women on the left (solid points) and men on the right (open points). The dashed lines are set at *r* = 0.10 and *r* = − 0.10 to indicate that values above or below the lines, respectively, reflect meaningful effects. Several features are worth highlighting. First, most effects for men are below *r* = − 0.10, consistent with the sample-level findings indicating that feminine-to-masculine decreases are associated with anxiety, depression, and self-reproach increases. Second, individuals tend to show consistent effects, that is, feminine-to-masculine correlations with anxiety, depression, and self-reproach are often similar in direction for a person, with some variations in magnitude. Third and perhaps most importantly, many effects for individuals do not follow the patterns detected for their gender in the sample-level analyses. This includes several men for whom internalizing correlations with feminine-to-masculine expression are positive (reflecting better adjustment with reduced gender typicality), many individuals whose correlations are between *r* = − 0.10 and *r* = 0.10 (reflecting an inconsequential yoking between daily gender expression and internalizing), and the lack of a consistent pattern for women (with feminine-to-masculine decreases associated with increases in problems nearly as often as they are associated with decreases in problems).

Results from analyses considering masculinity and femininity as separate dimensions are provided in the Supplementary Information. They are strikingly consistent with patterns seen in the feminine-to-masculine continuum. For men, increases in daily femininity and decreases in daily masculinity typify psychological adjustment problems (i.e., increased daily anxiety, depression, and self-reproach). For women, there were not consistent patterns, with links between masculinity, femininity, and adjustment being person-specific.

## Discussion

Findings from this 75-day intensive longitudinal study containing over 4000 daily reports from 57 self-identified men and women begin to fill critical knowledge gaps in science and society’s understanding of gender and its implications for psychological adjustment. Results show that gender expression (operationalized as a continuum of feminine-to-masculine self-perceptions) fluctuates meaningfully across days, and does so for women more than men, but that daily psychological consequences of gender non-typicality (e.g., increased femininity/reduced masculinity for men) are greater for men than they are women. Critically, however, these associations did not describe all participants: Sample-level inverse relations between daily gender expression and anxiety, depression, or self-reproach in men only characterized about half of individuals.

Hypotheses regarding daily fluctuation in gender expression were supported, with within-person variation being about a third (for men) to two-thirds (for women) as large as between-person variation. Women also had moderately greater fluctuations in daily gender expressions than did men. Although unanticipated, this gender difference aligns with a growing literature suggesting greater gender flexibility in women than men^44^. Interestingly, four individuals reported no daily fluctuations. This may reflect intentional reporting (e.g., self-directed gender policing), as two participants reported maximal sex-congruent scores every day. Finally, most daily fluctuations in gender expression were in gender-typical ranges, indicating that variation does not reflect “qualitative” shifts in gender identity, which shows a very large difference between men and women^45^.

Hypotheses regarding sample-level links between daily gender expression and psychological adjustment were partially supported in the multilevel models, as fixed effects revealed that the feminine-to-masculine continuum was inversely associated with anxiety, depression, and self-reproach for men, but that there were no significant associations for women. Crucially, findings for men provide a novel generalization of the trait-like associations between positive adjustment and gender typicality from other work^7,24,28–32^ to the daily (i.e., state-like) level. In other words, being masculine and “well-adjusted” does not just characterize who some men are, but it also characterizes their daily experiences: They feel good today because they perceive themselves to be less feminine and more masculine, but they will not feel so good tomorrow if their masculinity declines.

The null findings for women were counter to hypotheses, but it is reasonable that effects were greater in men for whom there are different (and often greater) societal consequences for violating gender norms^46^. Although there were no gender differences in overall levels of anxiety, depression, and self-reproach, null findings for women may also be related to the focus on female-typed internalizing^47^. Thus, psychological consequences of masculinity in women might become apparent if externalizing (i.e., male-typed adjustment) problems are assessed in future work.

Hypotheses regarding person-specific links were supported because the psychological adjustment consequences of daily fluctuations in gender expression differed substantially across people (consistent with the random effects in the multilevel models). Daily expression—though variable—did not have meaningful consequences (i.e., display at least a small effect of *r* >|.10|) in about 40% of the sample for any given internalizing outcome. Reductions in the feminine-to-masculine continuum were related to increases in internalizing in about 50% of men, with the implications of increases in the continuum varying notably across women. This person-specificity aligns with principles of gender multidimensionality^8^ as well as gender salience and beliefs, as individuals differentially attend to, process, and respond to gendered cues and contexts^48^. It also resonates with the more general concept of non-ergodicity^33^, or that sample-level findings do not equally apply to each individual. Thus, an important implication of this study is that, as gender and its links with psychological adjustment are non-ergodic, future research should utilize extensive within-person assessment methods that afford person-specific investigations and inferences^49^.

Findings from analyses in which gender expression was operationalized as a feminine-to-masculine continuum (main text) and in which masculinity and femininity were considered as separate dimensions (Supplementary Information) are wholly consistent. Effects were not driven by masculinity nor femininity alone, but rather were present in both dimensions (e.g., both decreased masculinity and increased femininity were linked to internalizing problems for about 40% of men). This was expected. It is consistent with current views of gender identity as largely being a single continuum^2,4^, and with state-of-the-science work using similar bipolar operationalizations of gender expression^1,3^; it also suggests gender self-perceptions may be an efficient way to assess both an individual’s identity and their knowledge of, and prescription to, sociocultural norms. These findings, therefore, encourage future work on the study of expressed gender self-concept as a relatively unidimensional continuum that maximizes measure validity and reliability as well as the statistical power of links with psychological outcomes^7,9^. This may be inconsistent with conclusions of classic work^6,14^ due to changes in societal gender norms in the last 40 + years or due to the multidimensionality of gender, as classic work considers gendered personal characteristics that may differ from gender expressions in sex-typed structure.

### Study considerations

Several features of the study design warrant comment. The between-person sample size may seem small, but gender expression is among the largest known sex differences^8,45^, and each participant provided up to 75 observations; thus, there was likely sufficient power to detect effects of interest, and effect sizes were consistently provided. Also, the sample consisted of young adults, did not include individuals across spectra of gender identities or clinical problems, and excluded individuals with known exogenous sex hormone alterations (i.e., oral contraceptive users) or missing data over 20%. Although these decisions enhance internal validity, they also limit generalizability. Future work in gender diverse samples is essential, and is now informed by methods and findings from a cisgender sample. Finally, though valid, reliable, and based on self-perceptions rather than sociocultural norms, the measure of gender expression (i.e., the Sex Role Identity Scale^10^) focuses solely on masculinity and femininity. Thus, it is important to reproduce or qualify this study’s pattern of findings using a measure that reflects gender expression as a continuum (e.g., with questions about non-binary daily appearance, actions, and feelings), consistent with current clinical conceptualizations^2,4^.

### Conclusions

Gender—and its expression—is typically thought to be stable, and research shows that it is related to psychological adjustment, such that individuals whose identities and biological sex misalign often report internalizing problems. Yet, the extant research is limited by single measurement occasions that describe the “average” man or woman. This is problematic because gender expression is not constant and does not mean the same thing for everyone. This study begins to fill this knowledge gap using innovative person-specific data collection and analytic methods. Between- and within-person analyses of 75-day intensive longitudinal data on gender expression (operationalized as a feminine-to-masculine continuum) and internalizing problems revealed significant daily fluctuations in gender for 93% of young adults. At the sample-level, these fluctuations were significantly related to anxiety, depression, and self-reproach only in men, such that daily increases in femininity or decreases in masculinity were associated with declines in adjustment. At the individual-level, however, these patterns did not describe about 50% of men, and relations varied across women. Importantly, daily fluctuations in gender expression were linked to internalizing problems in different ways for different people, highlighting the multidimensionality of gender and the pressing need for future person-specific work that leverages intensive longitudinal study designs.

## Supplementary Information

Supplementary Information.

## Data Availability

The data analyzed in this study are available from the corresponding author upon reasonable request.
