# Supplementary material for: Daily gender expression is associated with psychological adjustment for some people, but mainly men
Source: Sci Rep. 2021 Apr 27;11:9114. doi: 10.1038/s41598-021-88279-4 (PMC8079363; doi:10.1038/s41598-021-88279-4)
Supplement: Supplementary file 1 — Supplementary Information. [file 41598_2021_88279_MOESM1_ESM.pdf]

Supplemental Materials for "Daily gender expression is associated with psychological adjustment for some people, but mainly men"

Adriene M. Beltz, Amy M. Loviska, Alexander Weigard

Results

Daily Fluctuations in Masculinity

There was substantial fluctuation in daily masculinity, according to a one-sample *t*-test of iSDs, $t(56)=9.74$, $p<.001$, $d=1.30$, indicating that the sample average iSD of .39 ($SD=.30$) was greater than 0. Across individuals, masculinity iSDs ranged from 0 to 1.77, with 95% of the sample reporting fluctuation, but 3 individuals (1 woman and 2 men) reporting absolute stability, and an individual with an iSD of 1.77 being an outlier (i.e., three standard deviations beyond the mean). Removing this male participant did not affect inferences, as the one-sample *t*-test was still significant, $t(55)=11.37$, $p<.001$, $d=1.54$, and the sample average only reduced slightly to .37 ($SD=.24$). Individuals with no fluctuation and the outlier were excluded from the subsequent person-specific analyses (see below). Moreover, within-person variation in masculinity is nearly a third as large as the variation between people (i.e., standard deviation of masculinity across the sample): $M_{iSD}/SD_M=.37/1.24=.30$.

When considered separately, both men, $t(28)=8.49$, $p<.001$, $d=1.57$, and women, $t(26)=7.82$, $p<.001$, $d=1.52$, had significant fluctuation in daily masculinity. When comparing the genders, women ($M=.41$, $SD=.27$) and men ($M=.33$, $SD=.21$) did not differ in daily masculinity fluctuations, $t(54)=1.21$, $p=.233$, $d=.33$. Within-person variation in masculinity was a third as large as the between-person variation among men (.33/1.04=.32), but nearly two-thirds as large as the between-person variation among women (.41/.67=.61).

Daily Fluctuations in Femininity

There was substantial fluctuation in daily femininity, according to a one-sample *t*-test of iSDs, $t(56)=10.14$, $p<.001$, $d=1.34$, indicating that the sample average iSD of .43 ($SD=.32$) was greater than 0. Across individuals, masculinity iSDs ranged from 0 to 1.80, with 91% of the sample reporting fluctuation, but 5 individuals (1 woman and 4 men) reporting absolute stability, and an individual with an iSD of 1.80 being an outlier (i.e., three standard deviations beyond the mean); this was the same individual who was an outlier in masculinity fluctuation. Removing this male participant did not affect inferences, as the one-sample *t*-test was still significant, $t(55)=11.49$, $p<.001$, $d=1.52$, and the sample average only reduced slightly to .41 ($SD=.27$). Individuals with no fluctuation and the outlier were excluded from the subsequent person-specific analyses (see below). Moreover, within-person variation in masculinity is more than a third as large as the variation between people (i.e., standard deviation of femininity across the sample): $M_{iSD}/SD_M=.43/1.14=.38$.

When considered separately, both men, $t(28)=7.79$, $p<.001$, $d=1.50$, and women, $t(26)=9.16$, $p<.001$, $d=1.75$, had significant fluctuation in daily femininity. When comparing the genders, however, women ($M=.49$, $SD=.28$) reported significantly greater fluctuations than men ($M=.33$, $SD=.22$), $t(54)=2.50$, $p=.015$, $d=.64$. Within-person variation in femininity was nearly half as large as the between-person variation among men (.33/.72=.46), but slightly greater than that among women (.49/.81=.60).

Person-specific Links Between Daily Masculinity and Psychological Adjustment

Person-specific associations of masculinity with anxiety, depression, and self-reproach were quantified. For anxiety, correlations ranged from -.53 to .42 ($M=-.03$, $SD=.20$), with 56% meeting the threshold for a meaningful effect (i.e., $|r|>.10$). There was an average effect of -.09

across men that was below the negative threshold for 37% of men, but was above the positive threshold for another subset (17%). The average effect was approximately null in women (.04), with 37% reporting that masculinity increases co-occurred with anxiety increases, and 22% reporting co-occurring anxiety decreases. For depression, full sample correlations ranged from -.62 to .45 (*M*=-.06, *SD*=.21), with 56% meeting the threshold for a meaningful effect. In men, there was again a small average inverse effect (-.10) that typified 43% of individuals, but in women, the average effect was basically null (-.03) with 22% showing a positive masculinity-depression relation and a higher 30% showing an inverse relation. For self-reproach, full sample correlations ranged from -.64 to .67 (*M*=-.05, *SD*=.22), with 54% having a meaningful effect. In men, the small average inverse effect (-.06) typified a majority (43%) of individuals, and the average effect in women (-.04) was likely due to 19% showing a positive masculinity-self-reproach relation and a higher 26% showing an inverse relation.

Person-specific Links Between Daily Femininity and Psychological Adjustment

Person-specific associations of femininity with anxiety, depression, and self-reproach were quantified. For anxiety, correlations ranged from -.49 to .47 (*M*=.01, *SD*=.18), with 53% meeting the threshold for a meaningful effect (i.e., $|r|>.10$). There was an average effect of .05 across men that was below the positive threshold for 33% of men, but was above the negative threshold for another subset (17%). The average effect was approximately null in women (-.04), with 33% reporting that femininity decreases co-occurred with anxiety decreases, and 22% reporting co-occurring anxiety increases. For depression, full sample correlations ranged from -.57 to .49 (*M*=-.01, *SD*=.21), with 61% meeting the threshold for a meaningful effect. In men, the average positive effect (.03) typified 43% of individuals, but in women, the average effect (-.05) included 33% showing an inverse femininity-depression relation but 22% showing a positive

relation. For self-reproach, full sample correlations ranged from -.31 to .67 (*M*=.06, *SD*=.19), with 60% having a meaningful effect. In men, the positive effect (.11) again typified a majority (43%) of individuals, and the null effect in women (.005) was likely due to 30% showing an inverse femininity-self-reproach relation and a similar 33% showing a positive relation.

Table S1. *Multilevel model results for the sample-level link between daily masculinity and three indices of psychological adjustment by gender*

Outcome	Parameter or Fit Index	Unstandardized Estimate (SE) or Fit Statistic		
		**Full Sample**	**Men**	**Women**
**Anxiety**	*Fixed Effects*:			
	Intercept	2.73 (.13)***	2.86 (.12)***	2.73 (.13)***
	Day	-.0007 (.001)	-.0007 (.001)	-.0008 (.002)
	**Masculinity**	.10 (.05)	**-.21 (.05)*** **	.10 (.05)
	Gender	.14 (.17)	--	--
	**Gender* Masculinity**	**-.31 (.07)*** **	--	--
	Random Effects:			
	Intercept	.38 (.08)***	.37 (.11)***	.39 (.13)**
	Day	.00005 (.00001)***	.00004 (.00001)**	.00006 (.00002)**
	**Masculinity**	**.03 (.01)** **	**.03 (.02)* **	.03 (.02)
	Model Fit:			
	AIC	7886.41	3956.10	3928.64
**Depression**	*Fixed Effects*:			
	Intercept	2.46 (.12)***	2.77 (.11)***	2.45 (.12)***
	Day	-.0009 (.0008)	-.0006 (.001)	-.001 (.0009)
	**Masculinity**	-.004 (.06)	**-.22 (.06)** **	-.02 (.05)
	Gender	.31 (.16)	--	--
	**Gender* Masculinity**	**-.23 (.08)** **	--	--
	Random Effects:			
	Intercept	.29 (.07)***	.29 (.09)**	.32 (.12)**
	Day	.00002 (.00001)**	.00003 (.00001)**	.00001 (.00001)
	**Masculinity**	**.05 (.02)** **	**.07 (.03)* **	.03 (.02)
	Model Fit:			
	AIC	7635.11	3888.56	3751.51
**Self-Reproach**	*Fixed Effects*:			
	Intercept	1.96 (.12)***	2.28 (.12)***	1.94 (.11)***
	Day	.002 (.0009)	.0007 (.001)	.003 (.001)*
	**Masculinity**	.03 (.06)	-.13 (.08)	.02 (.04)
	Gender	.31 (.16)	--	--
	**Gender* Masculinity**	-.17 (.09)	--	--
	Random Effects:			
	Intercept	.31 (.07)***	.37 (.11)**	.27 (.09)**
	Day	.00004 (.00001)***	.00005 (.00002)**	.00003 (.00001)**
	**Masculinity**	**.07 (.02)*** **	**.14 (.05)** **	.02 (.01)
	Model Fit:			
	AIC	6443.50	3533.20	2914.00

Table S2. *Multilevel model results for the sample-level link between daily femininity and three indices of psychological adjustment by gender*

Outcome	Parameter or Fit Index	Unstandardized Estimate (SE) or Fit Statistic		
		Full Sample	Men	Women
**Anxiety**	*Fixed Effects*:			
	Intercept	2.69 (.13)***	2.83 (.14)***	2.68 (.15)***
	Day	-.0008 (.001)	-.0009 (.001)	-.0006 (.002)
	**Femininity**	-.07 (.05)	**.13 (.06)***	-.06 (.05)
	Gender	.13 (.18)	--	--
	**Gender* Femininity**	**.21 (.07)****	--	--
	Random Effects:			
	Intercept	.45 (.09)***	.47 (.14)**	.51 (.16)**
	Day	.00005 (.00001)***	.00005 (.00002)**	.00006 (.00002)**
	**Femininity**	**.04 (.01)****	**.06 (.03)***	**.03 (.01)***
	Model Fit:			
	AIC	7899.59	3983.92	3915.08
**Depression**	*Fixed Effects*:			
	Intercept	2.47 (.12)***	2.69 (.12)***	2.48 (.12)***
	Day	-.0009 (.0008)	-.0008 (.001)	-.0009 (.0010)
	**Femininity**	-.07 (.06)	.11 (.07)	-.06 (.05)
	Gender	.21 (.17)	--	--
	**Gender* Femininity**	.17 (.09)	--	--
	Random Effects:			
	Intercept	.34 (.07)***	.33 (.10)**	.35 (.12)**
	Day	.00002 (.00001)***	.00004 (.00001)**	.00001 (.00001)
	**Femininity**	**.07 (.02)****	**.11 (.04)****	.03 (.01)
	Model Fit:			
	AIC	7657.22	3918.69	3741.81
**Self-Reproach**	*Fixed Effects*:			
	Intercept	1.93 (.12)***	2.27 (.12)***	1.92 (.11)***
	Day	.002 (.001)	.0006 (.001)	.003 (.001)*
	**Femininity**	-.03 (.06)	**.17 (.08)***	-.04 (.03)
	Gender	.33 (.16)	--	--
	**Gender* Femininity**	**.19 (.08)***	--	--
	Random Effects:			
	Intercept	.33 (.07)***	.37 (.11)**	.29 (.09)**
	Day	.00005 (.00001)***	.00005 (.00002)**	.00004 (.00001)**
	**Femininity**	**.06 (.02)****	**.13 (.04)****	.01 (.01)
	Model Fit:			
	AIC	6464.73	3548.88	2909.78
